# Summary of Medical Science

**Published:** 1861

**Authors:** 


SUMMARY OF MEDICAL SCIENCE.—Edited by Walter S. Wells, M. D. Part I. New York: Charles T. Evans, Publisher.

Already favorably known to the profession as the compiler of an "*Epitome of* BRAITHWAITE'S RETROSPECT," and the "*Compendium of* RANKING'S ABSTRACT," Dr. Wells has placed the medical world under more weighty obligations by this, his recent contribution to our current literature. The work, as its name indicates, is a *Summary* of the most valuable articles published in "Braithwaite's," "Ranking's," the London, Dublin, Edinburg, French, German, and American Medical Journals of 1860, together with original contributions by leading men in their respective specialities. In its 300 pages, we have the very cream of the medical journalism of 1860, selected with enlightened judgment; properly arranged and classified, and furnishing, in itself, a complete digest of the improvements and status of Practical Medicine, Surgery, Midwifery, Toxicology, Chemistry and Materia Medica, for the past year; and so arranged in binding and paging as to facilitate the re-binding of each department separately, when the work becomes sufficiently voluminous.

The volumes are issued semi-annually, on 1st April and October, at $1.25 per number, or $2.00 per annum in advance. Address publisher as above.

